# Supplementary figures and images for: Genetic analyses led to the discovery of a super-active mutant of the RNA polymerase I
Source: PLoS Genet. 2019 May 28;15(5):e1008157. doi: 10.1371/journal.pgen.1008157 (PMC6555540; doi:10.1371/journal.pgen.1008157)

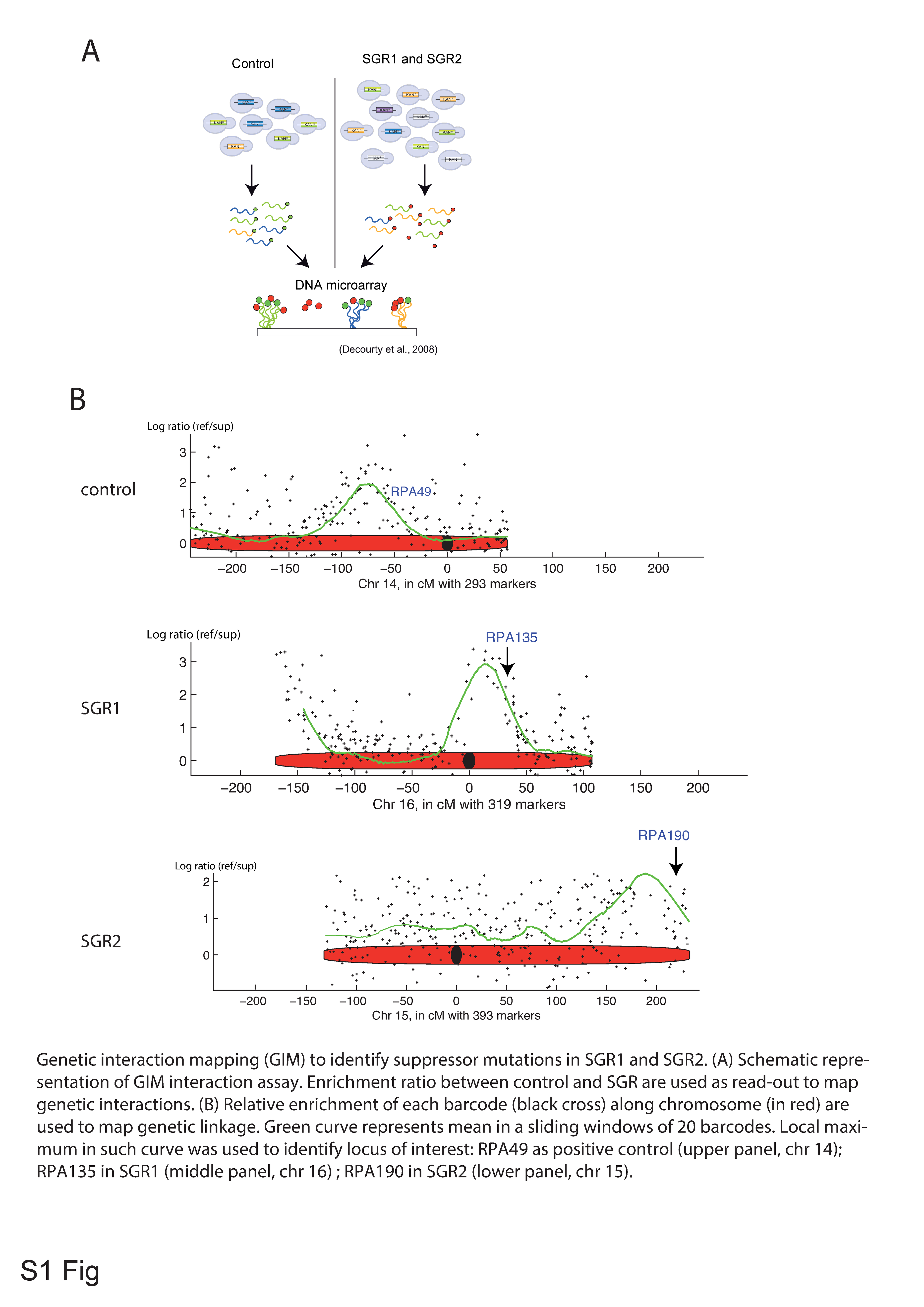

Supplement: S1 Fig — (A) Schematic representation of GIM interaction assay. Enrichment ratios between control and SGR are used as read-out to map genetic interactions. (B) Relative enrichment of each barcode (black cross) along chromosome (in red) is used to map genetic linkage. Green curve represents mean in a sliding windows of 20 barcodes. Local maximum in such curve was used to identify locus of interest: RPA49 as positive control (upper panel, chr 14); RPA135 in SGR1 (middle panel, chr 16); RPA190 in SGR2 (lower panel, chr 15). (TIF) [file pgen.1008157.s001.tif]

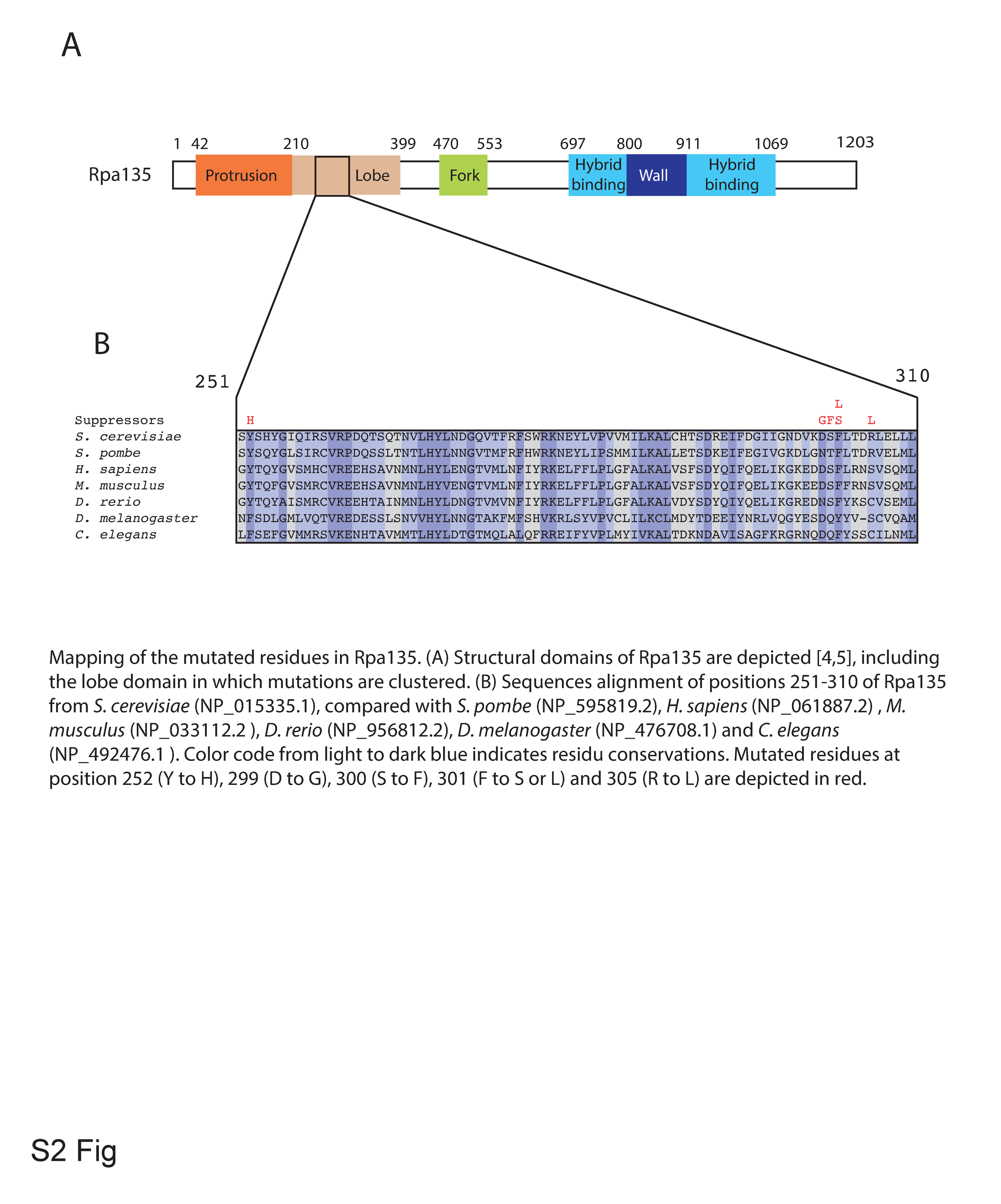

Supplement: S2 Fig — (A) Structural domains of Rpa135 are depicted [4,5], including the lobe domain in which mutations are clustered. (B) Sequences alignment of positions 251–310 of Rpa135 from S. cerevisiae (NP_015335.1), compared with S. pombe (NP_595819.2), H. sapiens (NP_061887.2), M. musculus (NP_033112.2), D. rerio (NP_956812.2), D. melanogaster (NP_476708.1) and C. elegans (NP_492476.1). Color code from light to dark blue indicates residue conservations. Mutated residues at position 252 (Y to H), 299 (D to G), 300 (S to F), 301 (F to S or L) and 305 (R to L) are depicted in red. (TIF) [file pgen.1008157.s002.tif]

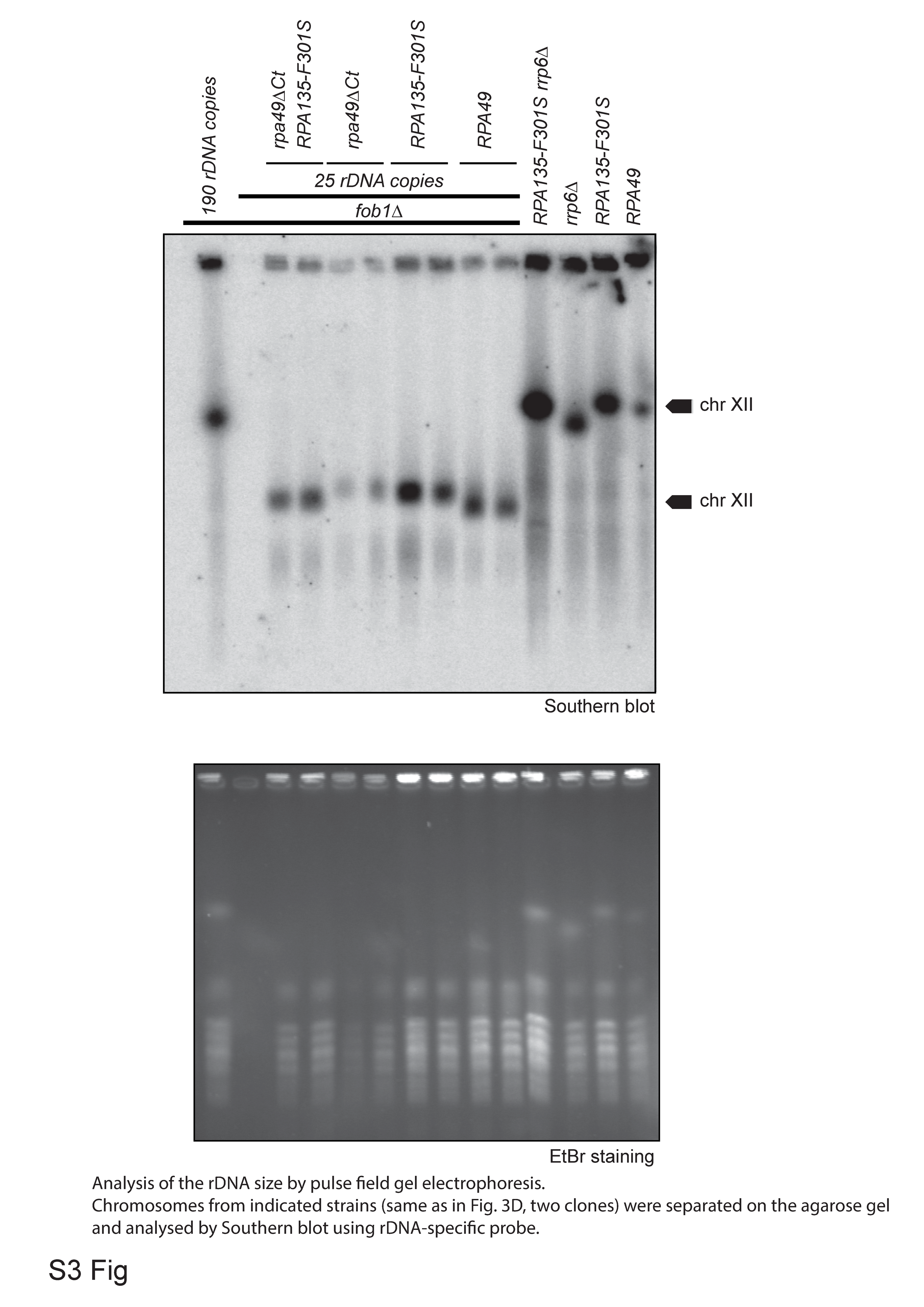

Supplement: S3 Fig — Chromosomes from indicated strains (same as in Fig 3D, two clones for rpa49ΔCt/RPA135-F301S; rpa49ΔCt; RPA135-F301S and RPA49) were separated on the agarose gel and analysed by Southern blot using rDNA-specific probe. (TIF) [file pgen.1008157.s003.tif]

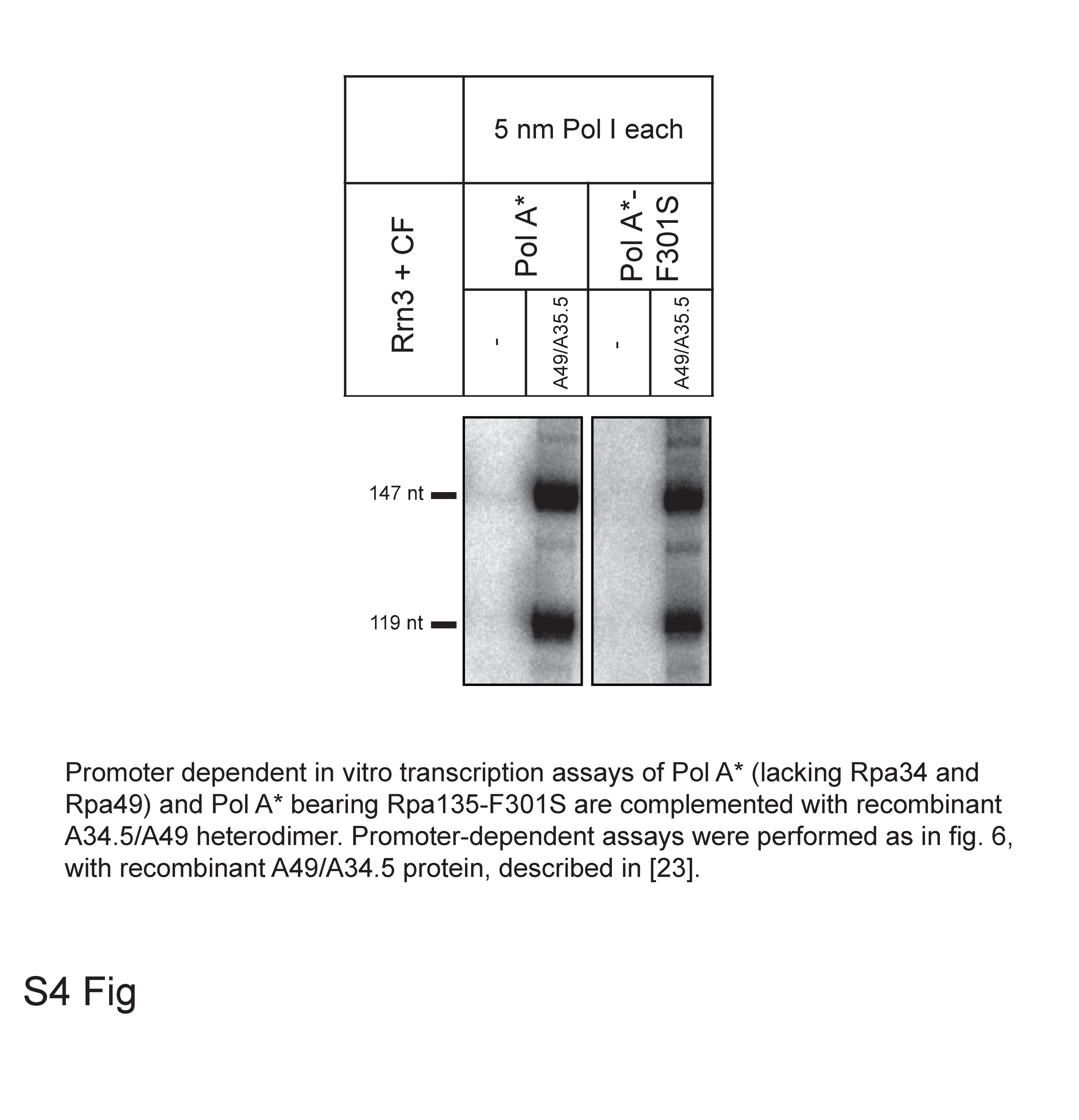

Supplement: S4 Fig — Promoter dependent in vitro transcription assays of Pol A* (lacking Rpa34 and Rpa49) and Pol A* bearing Rpa135-F301S are complemented with recombinant A34.5/A49 heterodimer. Promoter-dependent assays were performed as in Fig 6, with recombinant A49/A34.5 protein, described in [23]. (TIF) [file pgen.1008157.s004.tif]

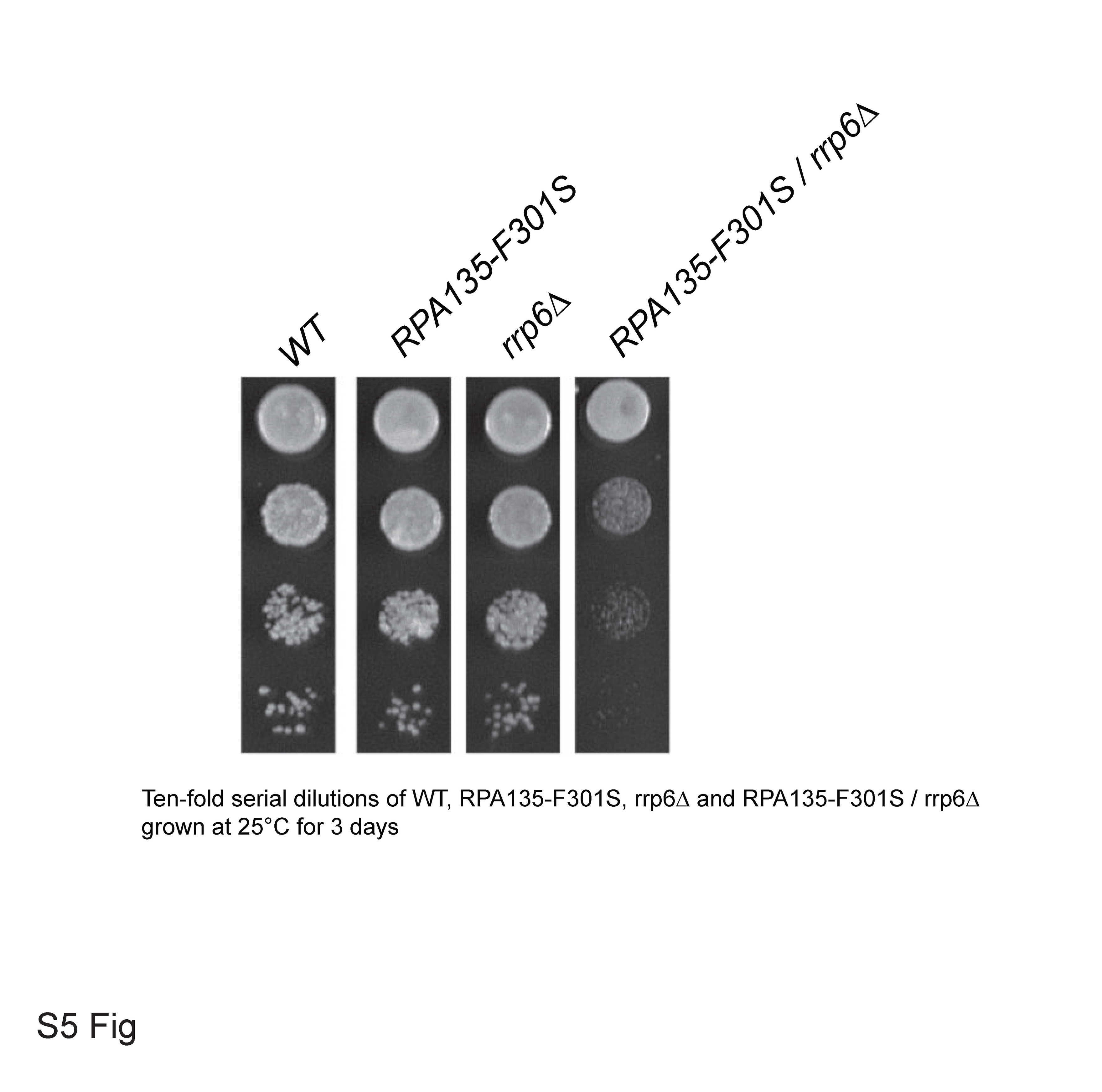

Supplement: S5 Fig — Ten-fold serial dilutions of WT, RPA135-F301S, rrp6Δ and RPA135-F301S -rrp6Δ grown at 25°C for 3 days. (TIF) [file pgen.1008157.s005.tif]

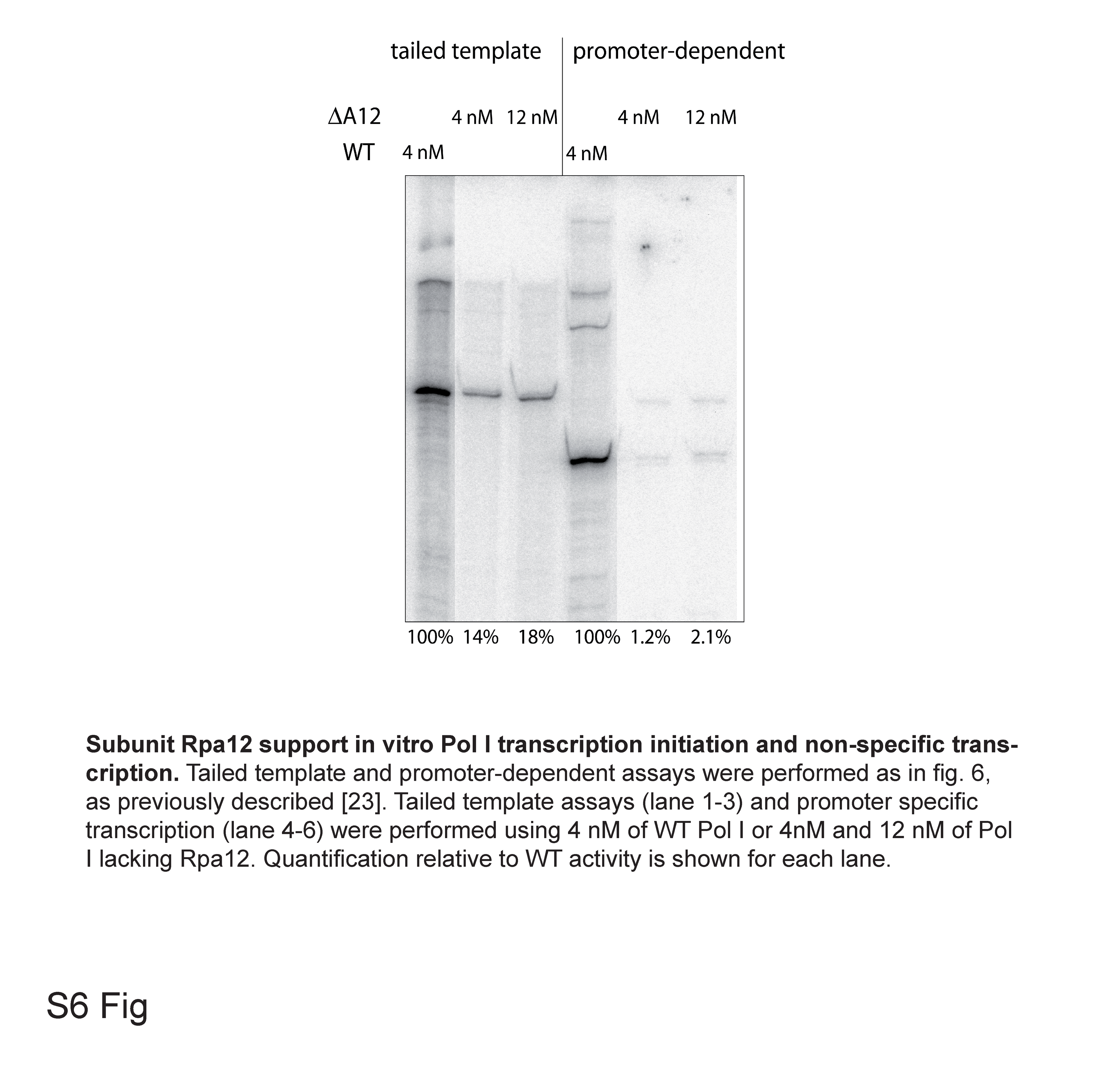

Supplement: S6 Fig — Tailed template and promoter-dependent assays were performed as in Fig 6, as previously described [23]. Tailed template assays (lane 1–3) and promoter specific transcription (lane 4–6) were performed using 4 nM WT Pol I or 4 nM and 12 nM of Pol I lacking Rpa12. Quantification relative to WT activity is shown for each lane. (TIF) [file pgen.1008157.s006.tif]
