# Supplementary material for: Genetic analyses led to the discovery of a super-active mutant of the RNA polymerase I
Source: PLoS Genet. 2019 May 28;15(5):e1008157. doi: 10.1371/journal.pgen.1008157 (PMC6555540; doi:10.1371/journal.pgen.1008157)
Supplement: S1 Table — (DOCX) [file pgen.1008157.s007.docx]

**S1 Table**

| **Sub.** | **Allele** | **Strength** | **Hotspot** | **Location** | **Mutant effect** |
| --- | --- | --- | --- | --- | --- |
| Rpa190 | N863T | Weak | Funnel | Funnel/ Rpa12-linker | Destabilization of Funnel/Rpa12 |
| Rpa190 | S1259L | Medium | Jaw | Jaw/Shelf hinge | Hinge conformation |
| Rpa190 | L1262P | Weak | Jaw | Jaw/Shelf hinge | Hinge conformation |
| Rpa190 | E1274K | Medium | Jaw | Jaw/ Rpa12-linker interface | Destabilization of Jaw/Rpa12 |
| Rpa190 | C1493R | Medium | Jaw | Jaw/ Rpa12-linker interface | Destabilization of Jaw/Rpa12 |
| Rpa135 | Y252H | Strong | Lobe | Jaw/Lobe interface | Destabilization of Jaw/Lobe |
| Rpa135 | D299G | Medium | Lobe | Jaw/Lobe interface | Destabilization of Jaw/Lobe |
| Rpa135 | S300F | Medium | Lobe | Jaw/Lobe interface | Destabilization of Jaw/Lobe |
| Rpa135 | F301S | Strong | Lobe | Jaw/Lobe interface | Destabilization of Jaw/Lobe |
| Rpa135 | F301L | Strong | Lobe | Jaw/Lobe interface | Destabilization of Jaw/Lobe |
| Rpa135 | **SGR3**  R305L | Medium | Lobe | Jaw/Lobe interface | Destabilization of Jaw/Lobe |
| Rpa12 | S6L | Medium | N-terminal | Jaw/ Rpa12-linker interface | Destabilization of Jaw/Rpa12 |
| Rpa12 | T49A | Medium | Linker | Jaw/ Rpa12-linker interface | Destabilization of Jaw/Rpa12 |
| Rpa190 | L608S | Medium |  |  |  |
| Rpa190 | E611K | Medium |  |  |  |
| Rpa190 | S936A | Weak |  |  |  |
| Rpa190 | **SGR2**  A1557V | Weak |  |  |  |
| Rpa135 | D157G | Medium |  |  |  |
| Rpa135 | D157N | Medium |  |  |  |
| Rpa135 | **SGR1**  I218/  R379K | Medium |  |  |  |
| Rpa135 | R379G | Strong |  |  |  |
| Rpa135 | G580D | Medium |  |  |  |
| Rpa135 | C584Y | Weak |  |  |  |
| Rpa135 | I913V | Medium |  |  |  |

List of 24 individual suppressor mutations of the growth defect of *rpa49∆* strain in the Rpa190, Rpa135, and Rpa12 subunits (Sub.). The suppressors were classified according to growth rate when combined with *rpa49∆* mutant: weak, medium, or strong. SGR1, 2, and 3 depict alleles originally isolated after UV mutagenesis (see text). Thirteen of the 24 mutants, affecting 12 different positions, were found in a specific hot-spot shown in Figure 4. Residues substitution in the 3D structure were performed *in silico*, and putative mutant effects were deduced from the obtained structure.
