# Supplementary material for: Genetic analyses led to the discovery of a super-active mutant of the RNA polymerase I
Source: PLoS Genet. 2019 May 28;15(5):e1008157. doi: 10.1371/journal.pgen.1008157 (PMC6555540; doi:10.1371/journal.pgen.1008157)
Supplement: S2 Table — (DOCX) [file pgen.1008157.s008.docx]

**S2 Table: Yeast strains used in this study**

| **Referred to as** | **strain** | **Genotype** | **source** |
| --- | --- | --- | --- |
| *WT in figures 1,4 and 7* | *BY4741* | *MATa his3∆1 leu2∆0 met15∆0 ura3∆0* | *Euroscarf* |
|  | *BY4742* | *MATalpha his3∆1 leu2∆0 lys2∆0 ura3∆0* | *Euroscarf* |
|  | *y1196* | *MATa his3∆1 leu2∆0 lys2∆0 ura3∆0 rpa49∆::KANMX4* | *Euroscarf* |
|  | *y27138* | *MATa/alpha his3∆1/his3∆1 leu2∆0/leu2∆0 ura3∆0/ura3∆0 lys2∆0/LYS2 MET15/met15∆0 rpa135∆::KANMX4/RPA135* | *Euroscarf* |
|  | *TGT135-3b* | *MATa his3∆11 leu2∆0 ura3∆0 lys2∆0 rpa135∆::KANMX4*  *+pNOY80* | *Euroscarf* |
|  | *TGT12* | *MATa/alpha his3∆1/his3∆1 leu2∆0/leu2∆0 ura3∆0/ura3∆0 lys2∆0/lys2∆0 rpa135∆::KANMX4/RPA135 rpa49∆::HPHMX4/RPA49* | *Euroscarf* |
| *rpa49∆ in figure 1, with the plasmids indicated in the legend* | *OGT9-6a* | *MATalpha his3∆1 leu2∆0 met15∆0 ura3∆0 rpa49∆::alphaNATMX6* | *This study* |
| *rpa49∆ in figure 4A, with the plasmids indicated in the legend* | *OGT8-11a* | *MATa his3∆1 leu2∆0 lys∆0 ura3∆0 rpa49∆::KANMX6* | *This study* |
| *SGR1 in figure 1* | *OGT15-7b* | *MATa his3∆1 leu2∆0 lys∆0 ura3∆0 HIS3::RPA135(I218T, R379K)* | *This study* |
| *SGR1 rpa49∆ in figure 1* | *LH514D* | *MATalpha his3∆1 leu2∆0 met15∆0 ura3∆0 rpa49∆-alphaNAT RPA135(I218T, R379K)* | *This study* |
|  | *AH29R* | *MATa his3∆1 leu2∆0 lys∆0 ura3∆ rpa49∆- kanmx4 RPA190-1557V* | *This study* |
| *SGR2* | *AC5-3b* | *MATalpha his3∆1 leu2∆0 lys∆0 ura3∆ rpa49∆-alphaNAT RPA190-1557V* | *This study* |
| *SGR3* | *LH11D* | *MATalpha his3∆1 leu2∆0 met15∆0 ura3∆0 rpa49∆-alphaNAT RPA135-R305L* | *This study* |
|  | *RPA135-TAP* | *MATalpha his3∆1 leu2∆0 met15∆0 ura3∆0 RPA135-TAP::HIS3MX6* | *202233825*  [54] |
|  | *yTD16-1a* | *MATa ade2-1 ura3-1 his3-11,15 trp1-1 leu2-3,112 can1-100 fob1∆::NAT-MX, rDNA copy no. ~25* | *This study* |
|  | *yTD6-6c* | *MATa ura3∆0 his3-Δ1 leu2-Δ0 lys2-Δ0 RPA135-F301S-TAP-HIS3* | *This study* |
| *rpa49∆ in figures 2, 4A, 5* | *OGT15-9d* | *MATalpha his3∆1 leu2∆0 lys∆0 ura3∆0 rpa49∆::HPHMX* | *This study* |
| *WT in figures 3* | *yTD27-1a* | *MATa ade2-1 ura3-1 his3-11,15 trp1-1 leu-3,112 can1-100 fob1∆::NAT-MX RPA135-TAP-HIS3, rDNA copy number ~25*  *+ pCJF4-LEU-GAL49* | *This study* |
| *RPA135-F301S in figure 3* | *yTD28-1a* | *MATa ade2-1 ura3-1 his3-11,15 trp1-1 leu-3,112 can1-100 fob1∆::NAT-MX RPA135-F301S-TAP-HIS3, rDNA copy number ~25, + pCJF4-LEU-GAL49* | *This study* |
|  | *yTD25-1a* | *MATa his3∆1 leu2∆0 met15∆0 ura3∆0 rpa49∆C(186-416)::KAN-MX4* | *This study* |
|  | *yTD11-1a* | *MATa his3∆1 leu2∆0 met15∆0 ura3∆0 rpa49∆C(186-416)::HPH-MX4* | *This study* |
| *rpa49∆Ct in figure 3* | *yTD29-1a* | *MATa ade2-1 ura3-1 his3-11,15 trp1-1 leu-3,112 can1-100 fob1∆::NAT-MX RPA135 -TAP-HIS3 rpa49∆C(186-416)::HPH, rDNA copy number ~25,*  *+ pCJF4-LEU-GAL49* | *This study* |
| *rpa49∆Ct*  *RPA135-F301S*  *in figure 3* | *yTD30-1a* | *MATa ade2-1 ura3-1 his3-11,15 trp1-1 leu-3,112 can1-100 fob1∆::NAT-MX RPA135-F301S-TAP-HIS3 rpa49∆C(186-416)::HPH, rDNA copy number ~25,*  *+ pCJF4-LEU-GAL49* | *This study* |
| *RPA12-S6L in figures 3* | *yTD31-1a* | *MATa ade2-1 ura3-1 his3-11,15 trp1-1 leu-3,112 can1-100 fob1∆::NAT-MX RPA135- TAP-HIS3 RPA12-S6L-KAN-MX, rDNA copy number ~25,*  *+ pCJF4-LEU-GAL49* | *This study* |
| *rpa49∆Ct RPA12-S6L in figure 3.* | *yTD23_1a* | *MATa ade2-1 ura3-1 his3-11,15 trp1-1 leu-3,112 can1-100 fob1∆::NAT-MX RPA135- TAP-HIS3 RPA12-S6L-KAN-MX rpa49∆C(186-416)::HPHMX, rDNA copy number ~25* | *This study* |

*Supplementary table 2 (continued)*

|  | *SCOC2260* | *Mata ade2 arg4 leu2-3,112 trp1-289 ura3-52*  *RPB6::TAP-K.L.URA3 rpa190-∆LOOP-KANMX* | *This study* |
| --- | --- | --- | --- |
| *rpa190∆loop*  *in figure 4A* | *yTD48-1a* | *MATa his3∆1 leu2∆0 lys∆0 ura3∆0 rpa190∆loop::KANMX* | *This study* |
| *rpa190∆loop RPA135-F301S in figure 4A* | *yTD51-2c* | *MATalpha his3∆1 leu2∆0 met15∆0 ura3∆0 lys2∆0 rpa190∆loop::KANMX RPA135-F301S-URA3Kl* | *This study* |
| *rpa190∆loop RPA135-F301S*  *rpa49∆ in figure 4A* | *yTD51-8a* | *MATa his3∆1 leu2∆0 lys2∆0 met15∆0 ura3∆0*  *RPA135-F301S-URA3Kl rpa49∆::HPHMX* | *This study* |
| *RPA135-F301S*  *rpa49∆ in figure 4A* | *yTD51-5a* | *MATa his3∆1 leu2∆0 lys∆0 ura3∆0 RPA135-F301S-URA3Kl rpa49∆::HPHMX* | *This study* |
| *rpa49∆ in figures 4B and 4C* | *yCN223-2a* | *MATalpha his3∆1 leu2∆0 met15∆0 ura3∆0 lys2∆0 rpa49∆::KANMX* | *This study* |
|  | *yTD2-3b* | *MATa ura3∆0 his3-Δ1 leu2-Δ0 lys2-Δ0 rpa135::KANMX,*  *pGL135_33 (ARS/CEN URA3 RPA135-F301S)* | *This study* |
| *rpa49∆*  *RPA135-F301S in figures 4B and 4C* | *yTD2-3d* | *MATa his3∆1 leu2∆ ura3∆0 lys2∆0 rpa135∆::KANMX rpa49∆::HPHMX + pGL135_33 (ARS/CEN RPA135-F301S)* | *This study* |
| *rpa34∆ in figure 4B* | *yCN224-1a* | *MATalpha his3∆1 leu2∆0 met15∆0 ura3∆0 lys2∆0 rpa34∆::KANMX* | *This study* |
| *rpa34∆*  *RPA135-F301S in figure 4B* | *yTD36-2b* | *MATalpha his3∆1 leu2∆0 ura3∆0 lys2∆0 rpa34∆::NATMX*  *RPA135-F301S::URA3* | *This study* |
| *rpa34∆ rpa49∆ in figure 4B* | *yTD37-7d* | *MATalpha his3∆1 leu2∆0 ura3∆0 lys2∆0 rpa34∆::NATMX*  *rpa49∆::HPHMX* | *This study* |
| *rpa34∆ rpa49∆*  *RPA135-F301S in figure 4B* | *yTD37-3d* | *MATalpha his3∆1 leu2∆0 ura3∆0 lys2∆0 rpa34∆::NATMX*  *rpa49∆::HPHMX RPA135-F301S::URA3* | *This study* |
| *rpa14∆ in figure 4C* | *yCN225-1a* | *MATalpha his3∆1 leu2∆0 lys∆0 ura3∆0 rpa14∆:: KANMX* | *This study* |
| *rpa14∆*  *RPA135-F301S in figure 4C* | *yTD38-3d* | *MATa his3∆1 leu2∆0 ura3∆0 lys2∆0 rpa14∆::NATMX*  *RPA135-F301S::URA3* | *This study* |
| *rpa14∆ rpa49∆*  *RPA135-F301S in figure 4C* | *yTD39-8a* | *MATa his3∆1 leu2∆0 ura3∆0 lys2∆0 rpa14∆::NATMX*  *rpa49∆::HPHMX RPA135-F301S::URA3* | *This study* |
|  | *yTD53-1a* | *MATalpha his3∆1 leu2∆0 lys2∆0 ura3∆0 KANMX6-pGAL::RPA12* | *This study* |
| *RPA12 alleles in figure 5 with the plasmids indicated in the legend* | *OGT30-1c* | *MATa his3∆1 leu2∆0 lys∆0 ura3∆0 KANMX-pGAL::RPA12* | *This study* |
| *rpa49∆ in figure 5 with the plasmids indicated in the legend* | *OGT30-3c* | *MATalpha his3∆1 leu2∆0 lys∆0 ura3∆0 rpa49∆:: HPHMX*  *KANMX-pGAL::RPA12* | *This study* |
|  | *OGT30-1a* | *MATa ura3-Δ0 his3-Δ1 leu2-Δ0 lys2-Δ0 rpa49Δ::HPH*  *RPA135-(F301S)-TAP-HIS3 KANMX-pGAL::RPA12* | *This study* |
|  | *OGT30-3a* | *MATa ura3-Δ0 his3-Δ1 leu2-Δ0 lys2-Δ0 RPA135-(F301S)-TAP -HIS3 KANMX-pGAL::RPA12* | *This study* |
|  | *yTD40-1a* | *MATa ura3-Δ0 his3-Δ1 leu2-Δ0 lys2-Δ0 RPA135-(F301S)-STOP-URA3 KANMX-pGAL::RPA12* | *This study* |
|  | *yTD41-1a* | *MATa ura3-Δ0 his3-Δ1 leu2-Δ0 lys2-Δ0 rpa49Δ::HPH*  *RPA135-(F301S)-STOP-URA3 KANMX-pGAL::RPA12* | *This study* |
| *RPA135-F301S in figure 5C* | *yTD42-1a* | *MATa his3∆1 leu2∆0 lys∆0 ura3∆0 RPA135-F301S::URA3*  *KANMX-pGAL::RPA12* | *This study* |
| *rpa49∆*  *RPA135-F301S*  *in figure 5C* | *yTD43-1a* | *MATalpha his3∆1 leu2∆0 lys∆0 ura3∆0 rpa49∆:: HPHMX*  *RPA135-F301S::URA3 KANMX-pGAL::RPA12* | *This study* |
| *rrp6∆ in figure 7 and S5* | *yCD2-2a* | *MATa his3∆1 leu2∆0 ura3∆ met15∆0 rrp6::NAT* | *This study* |
|  | *yMKS8-1a* | *MATa his3∆1 leu2∆0 ura3∆ met15∆0 rrp6::NAT KANMX6-pGAL::RPA49* | *This study* |
| *Rpa135-F301S rrp6∆ in figure 7 and S5* | *yMKS9-9d* | *MATa his3∆1 leu2∆0 ura3∆ rrp6::NAT RPA135-F301S-TAP-HIS3* | *This study* |
