# Supplementary material for: Genetic analyses led to the discovery of a super-active mutant of the RNA polymerase I
Source: PLoS Genet. 2019 May 28;15(5):e1008157. doi: 10.1371/journal.pgen.1008157 (PMC6555540; doi:10.1371/journal.pgen.1008157)
Supplement: S3 Table — (DOCX) [file pgen.1008157.s009.docx]

**S3 table: Plasmids used in this study**

| Name | Description | Source |
| --- | --- | --- |
| pUC19-HPH | *Plasmid bearing HPH-MX4* | [55] |
| pFA6-kanMX6 | *Plasmid bearing KAN-MX6* | [56] |
| pFA6a-KanMX6-GAL::3HA | *Plasmid used for GAL promoter insertion* | [56] |
| pFA6a-HA-KlURA3 | *Plasmid used for epitope switching* | [57] |
| pNOY80 | *Plasmid CEN6 ARS4, URA3, RPA135* | [58] |
| pVV190 | *Plasmid pFL44-A190 (2µ URA3 RPA190)* | [24] |
| pRS316 | *CEN6 ARS4, URA3* | [59] |
| pCR4-HIS3 | *HIS3* | [59] [60] |
| pGL190_3 | *(2µ URA3 RPA190-E1274K) selected from randomly mutagenized p*VV190 | This study |
| pGL190_11 | *(2µ URA3 RPA190- C1493R) selected from randomly mutagenized p*VV190 | This study |
| pGL190_23 | *(2µ URA3 RPA190- L1262P) selected from randomly mutagenized p*VV190 | This study |
| pGL135_6prim | *(CEN4 URA3 RPA135-R379G selected from randomly mutagenized p*NOY80 | This study |
| pGL135_54 | *(CEN4 URA3 RPA135-Y252H) selected from randomly mutagenized p*NOY80 | This study |
| pGL135_33 | *(CEN4 URA3 RPA135-F301S) selected from randomly mutagenized p*NOY80 | This study |
| pTD1_3b_135TAP | *Plasmid pNOY80 bearing RPA135-TAP-HIS3* *obtained by homologous recombination using a PCR-amplified fragment generated with oligos 835 and 836 and genomic DNA of strain 202233825 as template.* | This study |
| pTD2_6c_135TAP | *Plasmid pNOY80 bearing RPA135-F301S-TAP-HIS3 obtained by homologous recombination using a PCR-amplified fragment generated with oligos 835 and 836 and genomic DNA of strain 202233825 as template.* | This study |
| pTD5 | *Plasmid pTD1_3b_135TAP deleted of URA3 using NsiI and SdaI digestion and self-ligation.* | This study |
| pTD6 | *Plasmid pTD2_6c_135TAP deleted of URA3 using NsiI and SdaI digestion and self-ligation.* | This study |
| Ycp50-26 | *URA3 ARS/CEN RPA49* | [59] |
| pRS316-A12 | *pRS316 vector ligated with a PCR-generated fragment using oligos 1554 and 1555 and yeast genomic DNA as template cut BamHI-XbaI, and cloned at same site.* | This study |
| pRS316-A12-AvrII | *PCR mediated mutagenesis to introduce AvrII site in* pRS316-A12 using oligos 1714 and 1715. | This study |
| pRS316-A12-S6L | *S6L Allele of RPA12 isolated as a suppressor of the rpa49∆ growth defect, selected from a PCR-mediated random mutagenesis of* pRS316-A12-AvrII. | This study |
| pRS316-A12-S6L-KAN | *Plasmid obtained using* pRS316-A12-S6L *modified by homologous recombination using a PCR-amplified fragment generated with oligos 1682 and 1559 and* pFA6-kanMX6 *as template.* | This study |
| pRS316-A12-T49A | *T49A Allele of RPA12 isolated as a suppressor of the rpa49∆ growth defect, selected from a PCR mediated random mutagenesis of* pRS316-A12-AvrII. | This study |
| pRS316-A12-DCt | *Plasmid obtained using* pRS316-A12 *modified by homologous recombination using a PCR-amplified fragment generated with* oligos *1371 and 1559 and* pFA6-kanMX6 *as template.* | This study |
| pTD9 | *Plasmid obtained using* pRS316-A12-T49A *modified by homologous recombination using a PCR-amplified fragment generated with* oligos *1371 and 1559 and* pFA6-kanMX6 *as template.* | This study |
| pTD10 | *Plasmid obtained using* pRS316-A12-S6L *modified by homologous recombination using a PCR-amplified fragment generated with* oligos *1371 and 1559 and* pFA6-kanMX6 *as template.* | This study |
| pCJPF4 | *pFL36cII with LEU2 marker, CEN4, containing RPA49 coding region* | This study |
| pCJPF4-GAL49-1 | *Plasmid obtained using pCJPF4 modified by homologous recombination using a PCR-amplified fragment generated with* oligos *624 and 625 and* pFA6a-KanMX6-GAL::3HA *as template.* | This study |
| pMAX1 | *Plasmid including Pol I promoter used for in vitro assays* | [23] |
| pUC19tail_g-_601_elongated | *Plasmid used for tailed template* | [61] |
