# Supplementary material for: Genetic analyses led to the discovery of a super-active mutant of the RNA polymerase I
Source: PLoS Genet. 2019 May 28;15(5):e1008157. doi: 10.1371/journal.pgen.1008157 (PMC6555540; doi:10.1371/journal.pgen.1008157)
Supplement: S4 Table — (DOCX) [file pgen.1008157.s010.docx]

**S4 table: Oligonucleotides used in this study**

| Sequence | N° |
| --- | --- |
| gctgctttatagaaccatctagcaaaaaaaaaaaccagagcaaaccaaatgctcagtatatacctctaggtaactgaattcgagctcgtttaaac | 208 |
| aaagcagttgaagacaagttcgaa | 301 |
| gactctctccaccgtttgacg | 302 |
| ggtctgtgatgcccttagacg | 307 |
| agtttcacaagattaccaagacctctc | 308 |
| ggtggtaaattccatctaaagctaaatatt | 311 |
| cacgtactttttcactctcttttcaaa | 312 |
| ccactattgcagtcgttatcaaccttttgcactttatctagtagaattcgagctcgtttaaac | 624 |
| cactttcaatttcgatttcagaaacagaccttttcacggacattttgagatccgggtttt | 625 |
| catagtttctggcatgaccc | 649 |
| gaagaatcctggtagtatgg | 650 |
| tcgatgaattcgagctcgt | 700 |
| gtcttcaactgctttcgcat | 774 |
| gacgctgatgacatcgagtg | 835 |
| tgatgggcacatatgaagtc | 836 |
| catggcttaatctttgagac | 892 |
| aggttctaacgttaatgtttctcaa | 1189 |
| actttataataccctagctctctta | 1194 |
| aagtcacaattctccaatttaaaagtcgtcaccacgacggcagacgatgcgtttccatcttctcttagagccaagaaatgaggcgcgccacttctaaa | 1371 |
| ccatcgaaagttgatagggcag | 1501 |
| ctacgttcgacttatacaaaaaaaagaagtctgaaaaggacgaattcgtattacacggtgaaaacgagagactagaataccggatccccgggttaattaa | 1515 |
| gggggatcctttcaaatattgctataaaaatggatgatagc | 1554 |
| cgctctagacactgaatgtcacgatagagttatc | 1555 |
| cactgaatgtcacgatagagttatcgctgt | 1556 |
| tttcaaatattgctataaaaatggatgata | 1557 |
| tctatagatgttcacatgatgaaagcggggatgatattaatgtacaaattgtaatatgtgcgaacacaacccaatcagaattcgagctcgtttaaac | 1559 |
| ctaatacggcattaggattttccaggagatcaccacagtccaagcaaaaaattaacgatcctacaacagacattttgagatccgggtttt | 1634 |
| tttatgtcaagtgaagggcattgcatggcaagtgtaaagaatccaagaaggaattcgagctcgtttaaac | 1635 |
| agttaagatctgcagatgaaggtgctactgtcttctatacatgcacttcctgtggttacaagttccgtaccaacaattgatgaggcgcgccacttctaaa | 1682 |
| ttgaagtacttggactctgagctatccgcaatgggtataagattgcgttataatgtagagcccaaataacggatccccgggttaattaa | 1679 |
| gatctcctggaaaatcctaatgccgtcctaggctctaacgttgaatgcagcc | 1714 |
| ggctgcattcaacgttagagcctaggacggcattaggattttccaggagatc | 1715 |
| gatttgtttaacggcttctttgtagtcatcctgctgttgtttttgtatgtttaagcttcttcctccgattaagttttgtacatgtaaaacgacggccagt | 1716 |
| tgatgcttttttgaagttttcatggcatgatttagcatttgaaatataatgagaaaagagccctttaacttacagaagtaaggaaacagctatgaccatg | 1717 |
| ttcttgcatgaacactaatgatattaggagggctttttaagttgctaccagaattcgagctcgtttaaac | 1711 |
| cactttcaatttcgatttcagaaacagaccttttcacggacatgcactgagcagcgtaatctg | 1713 |
| ctccgcttattgatatgc | 1829 |
| tgagaaggaaatgacgct | 1830 |
| accgtttggtctacccaagtgagaagccaagaca | 1831 |
| atcccggccgcctccatcac | 1832 |
| cggttttaattgtccta | 1833 |
| cctacagcgtgagctatgagaaag | 1834 |
| tcaccttaccctatacttactcg | 1835 |
| aaatggcctatcggaatacattttctacatcctaactactataaaacaacctttagacttacgtttgctactctcatggt | 1855 |
| tgcgaccggctattcaacaaggcattcccccaagtttgaattctttgaaatagattgctattagctagtaatccaccaaa | 1857 |
| ggaattcctcgttgaagagcaataattacaatgctctatccccagcacgacggagtttcacaagattaccaagacctctc | 1859 |
| gtgctggcctcttccagccataagaccccatctccggataaaccaattccggggtgataagctgttaagaagaaaagata | 1860 |
| gtaaatggtacactcttacacactatcatcctcatcgtatattataatagatatatacaatacatgtttttacccggatc | 1861 |
| tccgggcaaatcctttcacgctcgggaagctttgtgaaagcccttctctttcaacccatctttgcaacgaaaaaaaaaaa | 1862 |
| cagcttaactacagttgatcggacgggaaacggtgctttctggtagatatggccgcaaccgatagtttaacggaaacgca | 1863 |
| aaaaaaaaaaaagaaataaagattgcagcacctgagtttcgcgtatggtcacccactacactactcggtcaggctcttac | 1864 |
